# The cannabis-mind connection: a clinician’s perspective on mental health practice, harm reduction, and racial equity

**DOI:** 10.1186/s42238-025-00360-1

**Published:** 2025-11-22

**Authors:** Paulette S. Smith, Leah Sera

**Affiliations:** 1 Morgan State University, 1700 East Cold Spring Lane, Baltimore, MD USA; 2 University of Maryland School of Pharmacy, 20 N Pine St, Baltimore, MD USA

**Keywords:** Cannabis, Mental health, Harm reduction, Racial equity, Clinical stigma, Trauma-informed care

## Abstract

**Background:**

Cannabis is increasingly part of the mental health landscape, yet clinical practice has not kept pace with patient use. Many individuals rely on cannabis for anxiety, trauma, and mood concerns, often without professional guidance. Persistent stigma, limited training, and the racialized legacy of prohibition create barriers that prevent open dialogue in therapy and undermine patient trust.

**Main body:**

This perspective highlights the disconnect between patient experiences and clinical engagement with cannabis. Drawing on historical context, clinical insight, and emerging evidence, it considers how systemic inequities and professional discomfort shape current practice. Black and Brown communities have borne disproportionate harms from cannabis criminalization, influencing how cannabis use is perceived and discussed within care settings.

A harm reduction and person-centered approach provides a framework for clinicians to engage with patients in a more ethical and effective manner. This includes acknowledging cannabis use without judgment, exploring patients’ intentions and experiences, and supporting informed decisions about risks and benefits. Cultural humility and awareness of cannabis’s historical and therapeutic roles are essential to counteracting stigma and building safety in the therapeutic relationship. By moving beyond abstinence-only models, clinicians can foster transparency and trust while respecting patient autonomy.

**Conclusion:**

Clinicians need not endorse cannabis to engage meaningfully with patients. What is required is openness, informed practice, and recognition of the structural forces that shape patient behavior and disclosure. Approaching cannabis use as a valid subject of clinical conversation advances justice-oriented care and creates opportunities for more responsive and inclusive mental health practice.

## Positionality statement

As the principal author of this perspective, I write as a Black woman clinician, cannabis science and therapeutics educator, social work educator, and community advocate whose personal and professional experiences intersect with the legacy of cannabis prohibition and the pursuit of equitable mental health care. My positionality informs my approach to harm reduction and justice-oriented practice, centering the voices of Black and Brown individuals whose relationships with cannabis have been shaped by both healing and criminalization. My work reflects a commitment to cultural humility, integrative wellness, and clinicians' ethical responsibility to confront systemic and internalized bias in care.

As a contributing author to this perspective, I write as a White American (U.S.) woman, palliative care clinician, and medical cannabis educator. My understanding of the effects of cannabis criminalization and harm-reduction practices in cannabis medicine on Black and Brown communities in the U.S. has been cultivated primarily through academic research and discussion. Within the context of my clinical practice and teaching roles, I strive to practice cultural humility and to become a more effective advocate for health and social equity.

## Background

Cannabis use is increasingly part of the mental health landscape, with many individuals turning to it for anxiety, post-traumatic stress disorder (PTSD), depression, and sleep-related concerns (Walsh et al. 2017; Hindocha et al. 2020; Turna et al. 2017). Surveys show higher rates of cannabis use among people with mental health conditions compared to the general population, reflecting unmet treatment needs and gaps in care (Lev-Ran et al. 2014). For some patients, cannabis represents a first-line approach to symptom management; for others, it becomes a tool when conventional treatments feel inaccessible, unhelpful, or overly clinical in nature (Reid 2023).

Despite these realities, many mental health professionals remain hesitant or unprepared to address cannabis in practice. Research identifies persistent barriers —including stigma, ambiguous public health guidance, and insufficient training —across various health professions (Gardiner et al. 2019; Gali et al. 2020; Bosley et al. 2023). These challenges contribute to silence in clinical spaces, where patients often do not disclose cannabis use or encounter judgment when they do (King et al. 2024).

The roots of this silence extend beyond professional discomfort. The racialized history of cannabis prohibition has profoundly shaped how cannabis is perceived, legislated, and policed. Black and Brown communities have been disproportionately criminalized, with consequences that reverberate through families, communities, and healthcare systems (Bonnie and Whitebread 1970; 2020; Hansen et al. 2022; Bender 2023). This legacy fuels mistrust of providers and creates structural barriers to safe, open conversations about cannabis in mental health care (Rosino and Hughey 2018).

This Perspective aims to offer a clinician’s view on how mental health professionals can ethically and effectively address cannabis use in therapeutic practice. Drawing on clinical experience, contemporary evidence, and a narrative review of the literature, it explores the intersections of mental health care, harm reduction, and racial equity. Rather than debating whether cannabis is inherently harmful or beneficial, the issue under discussion is how clinicians can responsibly support patients who are already using cannabis while addressing stigma, inequities, and the structural forces that shape care.

### Equity and the legacy of prohibition

At the heart of cannabis stigma lies a long and painful history of racialized policy and systemic exclusion. It is impossible to talk about cannabis, especially in therapeutic contexts, without talking about race, history, and power. The legacy of prohibition is not just about the plant. It is about the people who have been harmed, excluded, and criminalized as a result of cannabis perceptions and policies (King et al. 2024; Hansen et al. 2022). Black and Brown communities have borne the brunt of cannabis-related arrests, surveillance, and stigma, even as others now profit from legalization (Bonnie and Whitebread 1970).

The War on Drugs, a set of state and federal policies and practices involving militarized policing and punitive sentencing, enacted under the guise of drug control in the latter half of the twentieth century, entrenched a mindset of punishment in policing and across education, housing, healthcare, and social services (Bender 2023). Helena Hansen and colleagues call this the “diffusion of drug war logic” across key social determinants of health (Hansen et al. 2022). This approach disproportionately targets people of color and systematically erodes trust in systems that are supposed to protect and heal.

#### Structural barriers

Racialized cannabis policies have disproportionately harmed Black, Brown, and immigrant communities (Bonnie and Whitebread 1970; Hansen et al. 2022). These same communities now face systemic barriers to accessing medical cannabis, equitable licensing, and culturally competent care (Rosino and Hughey 2018; Valencia et al. 2017). In the author’s experience, clients worry that even mentioning cannabis in therapy will lead to surveillance or custody concerns. Their caution is justified by decades of over-policing and social service involvement in their communities. These barriers are not only institutional; they are embodied. Patients carry intergenerational trauma from drug war policies, and they bring that history into the room, whether or not it is named. As Hansen et al. argue, this trauma manifests through diminished access to supportive care and fear of punitive consequences, particularly in Black and Brown families navigating behavioral health systems (Hansen et al. 2022).

Additionally, cannabis has held important roles in healing and spiritual practices across cultures. As Cousijn et al. describe, cannabis use is often embedded in community rituals and traditional medicine, complicating the dominant narrative of cannabis as merely a recreational or pathological substance (Cousijn 2023). When medical systems fail to recognize these practices, they exclude people and the cultural frameworks supporting well-being.

These structural inequities are compounded by implicit bias within healthcare settings. Studies show that clinicians’ racial assumptions lead to measurable disparities in care. For instance, Black patients are frequently undertreated for pain due to false beliefs about biological differences (Hoffman et al. 2016). Additionally, Black pregnant patients are disproportionately subjected to urine toxicology testing during labor and delivery, even without a documented history of substance use (Jarlenski et al. 2023). These findings make clear that bias is not an abstract concept but a quantifiable determinant of health—one that shapes how safety, credibility, and compassion are extended within clinical practice.

Within this context, Black and Brown women face compounded stigma that intensifies both their mental health challenges and their fear of disclosure. As Crenshaw first articulated through intersectionality theory, Black women occupy overlapping identities that expose them to simultaneous racial and gendered discrimination often obscured in single-axis analyses (Crenshaw 1989). Their cannabis use, in particular, is judged through intertwined narratives of race, gender, and motherhood. Decades of policy rooted in the War on Drugs have criminalized Black women’s survival and self-care practices, framing them as unfit mothers and moral failures rather than individuals coping with trauma (Simmons 2018). These historical legacies persist in the present, as societal narratives continue to pathologize Black women’s coping behaviors while questioning their capacity for caregiving and self-regulation (Faber et al. 2023). The result is a pervasive climate of fear and mistrust that discourages open discussion of cannabis use in therapy and other care settings. A justice-oriented approach therefore requires clinicians to understand how intersecting identities shape risk and resilience and to create spaces where Black women’s healing practices—including the use of cannabis for symptom relief or spiritual connection—are met with empathy rather than suspicion.

#### The clinician’s role in justice-oriented care

Mental health professionals have a responsibility to:

- Acknowledge cannabis criminalization's racialized history
- Validate patient fears around disclosure and legal risk
- Understand trauma's role in substance use
- Respect cultural and ancestral cannabis practices

Justice-oriented care begins with cultural humility (Tervalon and Murray-García 1998). For some patients, cannabis is more than a substance—it is part of community, resistance, and healing. Clinicians should be willing to see it through that lens. By naming the history, validating lived experiences, and supporting informed choice, clinicians dismantle stigma and co-create a space where healing is possible.

Justice-oriented care also requires introspection. Clinicians must confront implicit biases and cognitive dissonance—the internal tension that occurs when values of equity clash with unexamined assumptions that often influence perceptions of patients who use cannabis. Research shows that bias—both conscious and unconscious—can influence diagnostic accuracy, pain assessment, and treatment recommendations, leading to unequal outcomes for patients of color (Hoffman et al. 2016; Hagiwara et al. 2020). Confronting these biases involves tolerating discomfort, recognizing one’s role in systemic inequity, and engaging in ongoing self-assessment. Cognitive dissonance can be a powerful motivator for growth. Justice-oriented clinicians use this discomfort as information, not evidence of failure, and commit to unlearning racialized narratives embedded in professional training. Reflective supervision, anti-racism education, and honest dialogue within professional communities are vital steps in this process (Penner et al. 2010; Galvan and Payne 2024).

Frameworks such as Empowerment Theory and Critical Race Theory (Zimmerman 2000; 2017) reinforce this approach by addressing systemic power dynamics and promoting patient autonomy in care.

### Cannabis for mental health—a historical practice

While modern practice remains influenced by stigma and systemic bias, cannabis has long been used for healing across cultures. Understanding this history helps reclaim its therapeutic potential and reframe how we approach it in mental health care. Historically, cannabis was integrated into traditional health systems to address distress, disconnection, and imbalance—conditions central to mental health care (Russo 2007; Zuardi 2006).

In ancient China, cannabis was documented in the *Shennong Ben Cao Jing* as a remedy for worry and forgetfulness (Li 1974). In India, it was used in Ayurvedic medicine to calm the nervous system and aid spiritual connection (Russo 2007; Crocq 2020). In 19th-century Western medicine, cannabis tinctures were commonly prescribed for melancholia, insomnia, and nervous disorders. Sir John Russell Reynolds endorsed cannabis as a treatment for emotional instability (Reynolds 1890).

This therapeutic legacy was interrupted by racially motivated policy shifts in the United States (U.S.) The 1937 Marihuana Tax Act and the 1970 Controlled Substances Act criminalized cannabis, delegitimizing its medical use and reinforcing stereotypes about marginalized communities (King et al. 2024; Sloman 1998). As a result, cultural knowledge surrounding the cannabis plant was systematically erased. This history challenges the notion that cannabis is a "new" intervention. Reclaiming it as a mental health tool is about restoring autonomy and honoring cultural wisdom.

### Clinical evidence and contemporary use

After exploring the historical and systemic roots of cannabis stigma, clinicians need to consider what current research reveals about its role in mental health care. While often debated in extremes, either as a treatment breakthrough or a harmful crutch, (Bosley et al. 2023) most patients fall in between. They turn to cannabis not out of recklessness but as a response to unmet needs, inaccessible care, or ineffective treatments. Clinical evidence offers guidance but cannot replace the lived reality of each person’s experience.

#### Anxiety and mood disorders

Patients frequently report using cannabis to regulate mood and manage anxiety. While low-dose tetrahydrocannabinol (THC) and cannabidiol (CBD)-dominant products have shown anxiolytic effects, THC at higher doses can induce anxiety and paranoia (Crippa 2010; Hindocha et al. 2020). CBD, in contrast, demonstrates a safer side-effect profile and may offer therapeutic potential for social anxiety and generalized anxiety (Turna et al. 2017; Bergamaschi et al. 2011). It is not uncommon for patients to adjust their cannabis regimen on their own based on trial and error, figuring out which products worsen symptoms and which seem to help.

#### Depression

The evidence regarding cannabis and depression is mixed. Some users report mood elevation or emotional numbing, but longitudinal studies suggest heavy cannabis use may increase the risk of depression, especially in adolescents (Lev-Ran et al. 2014). The relationship may be bidirectional, with depressive symptoms motivating cannabis use as a form of self-medication (Turna et al. 2017). Clients may use cannabis to escape numbness, not to create it, seeking connection or creativity when they feel stuck.

#### Post-Traumatic Stress Disorder (PTSD) and trauma

Emerging data support cannabis as a symptom management tool for PTSD. Studies show reductions in hyperarousal, nightmares, and anxiety (Greer et al. 2014; Roitman et al. 2014). Black et al. note that individual studies have shown that pharmaceutical THC:CBD improves global functioning in PTSD (Black et al. 2019). While research is limited, the neurobiology of the endocannabinoid system suggests therapeutic potential for trauma-affected patients, especially when integrated into broader treatment plans (Marsicano et al. 2002). In therapeutic work, it becomes evident how profoundly trauma resides in the body and how cannabis, for some, gently softens that edge just enough to engage in healing.

#### Practice implications

Drawing on Bosley et al., clinicians are encouraged to move beyond binary thinking that either promotes or prohibits cannabis use (Bosley et al. 2023). Instead, they advocate for an informed, patient-centered approach that carefully considers how cannabis aligns with a patient’s health goals, current conditions, and safety. This includes actively listening, inquiring about form and dosage, and co-creating care plans that situate cannabis use within a broader context of wellness.

### Harm reduction in clinical settings

As clinicians begin to re-engage with cannabis as a therapeutic tool, many are turning to harm reduction as a guiding framework. This approach bridges clinical evidence with real-world use, centering safety, respect, and autonomy. In the evolving landscape of cannabis use and mental health care, harm reduction has emerged as a critical framework for clinicians seeking to provide ethically grounded, patient-centered treatment (Stone and Sherman 2023). Rather than positioning cannabis use as a barrier to care, harm reduction offers a pragmatic approach that recognizes patients’ autonomy and lived experiences while actively working to reduce potential harm (Denning and Little 2012; Sherman et al. 2022). This framework challenges the limitations of abstinence-only models, which often fail to address the nuanced reasons individuals use cannabis, particularly for managing mental health symptoms, trauma, and chronic stress (Gardiner et al. 2019; King et al. 2024). By emphasizing collaboration over control, harm reduction fosters more honest therapeutic relationships and supports incremental change, even in continued use (Denning and Little 2012; Swift et al. 2000).

#### Ethical engagement

Instead of insisting on abstinence, clinicians are encouraged to provide accurate information, explore usage patterns, and assess risks such as potency, co-use with alcohol, and method of administration (Greer et al. 2014). This approach reflects a public health commitment to helping individuals make safer, more informed choices, and it honors the therapeutic alliance by meeting patients where they are (Mathre 2002).

Still, stigma remains a powerful force within the provider-patient relationship. Clients may be afraid to disclose their cannabis use out of fear it will derail their care or have been told they weren’t “serious” about therapy unless they stopped using altogether. That kind of framing reinforces shame and ignores the reasons patients reached for cannabis in the first place, whether to manage sleep, regulate mood, or address trauma.

Recent findings confirm the widespread and harmful nature of these dynamics. In a national study of U.S. cannabis users, anticipated stigma, the expectation of being judged or treated differently by healthcare providers, was significantly associated with nondisclosure of cannabis use (King et al. 2024). This fear was the highest-rated stigma domain, surpassing enacted or internalized stigma. Notably, fewer than 15% of healthcare providers initiated discussions about cannabis use, while over half of patients had to raise the topic themselves. Nearly 28% reported that cannabis was never discussed at all.

Participants emphasized that disclosure was more likely when they felt safe and respected by their provider. Many wanted to share their cannabis use not just for safety reasons, but to be transparent and help their provider understand their complete health picture. Still, those who disclosed often encountered judgment or discomfort, reinforcing a reluctance to be open in future clinical settings. These experiences underscore the emotional labor patients must do to navigate a healthcare environment that has not yet normalized cannabis as part of therapeutic dialogue.

A harm reduction stance helps correct this imbalance. It creates space for transparency, reduces shame, and fosters trust (King et al. 2024; Denning and Little 2012). It does not require clinicians to condone or recommend cannabis, but to remain curious, respectful, and informed (Stone and Sherman 2023). Ultimately, this stance advances ethical care by prioritizing collaboration over control and reducing the harm caused by substances and the systems that stigmatize them (Gardiner et al. 2019; Hansen et al. 2022).

#### Practical strategies

When clinicians adopt a harm reduction approach, patients often feel more empowered and engaged in their care. This therapeutic orientation fosters trust and collaboration, allowing patients to explore their relationship with cannabis without fear of judgment or dismissal. As Denning and Little emphasize, harm reduction psychotherapy prioritizes patient autonomy and seeks to enhance motivation for change by building safety, curiosity, and self-reflection within the clinical relationship (Denning and Little 2012). In such environments, patients may independently adjust their usage or seek guidance because they trust the space is non-coercive and centered on their well-being. Even small shifts in cannabis use can significantly improve therapeutic alliance and overall functioning. As Sherman et al. note, modest reductions in use can yield measurable gains in mental health outcomes, making harm reduction a meaningful clinical success (Sherman et al. 2022).

In practice, harm reduction includes:

- Asking about cannabis use during routine assessments—not as a red flag, but as relevant clinical information (Sherman et al. 2022).
- Exploring intentions and functions of use without judgment (Denning and Little 2012).
- Educating patients on THC/CBD ratios and forms of consumption (Bosley et al. 2023; Crippa 2010; Bergamaschi et al. 2011).
- Promoting safer use practices (Set limits on how it is smoked and smoke in a safe environment with trusted friends who can offer support and reassurance.) (Sherman et al. 2022).
- Addressing polysubstance use risks through open, non-punitive dialogue (King et al. 2024; Stone and Sherman 2023).

### Bias-reduction as a core harm-reduction practice

Harm reduction is not only about substance use—it is also about reducing the harms created by stigma and inequity within clinical relationships. To operationalize justice-oriented care, clinicians must deliberately identify, unlearn, and interrupt bias in practice. These actions transform good intentions into accountable behaviors and model the same self-reflection clinicians seek to foster in their patients.

Concrete strategies include:

- Engage in ongoing anti-racism and implicit bias training. Continuing education should include the history of medical racism, the War on Drugs, and how structural inequities manifest in modern clinical decision-making (Simmons 2018; Faber et al. 2023; Bergkamp et al. 2022).
- Reflect on racial countertransference. Supervision and consultation should include honest exploration of how clinicians’ emotions, assumptions, or fears influence treatment planning and rapport.
- Solicit feedback from patients and colleagues. Structured reflection and peer dialogue can expose subtle inequities in tone, time allocation, or empathy that often go unnoticed (Tervalon & Murray-García, 1998; Penner et al., 2010).
- Integrate bias awareness into treatment documentation and supervision. Encourage journaling or supervision notes that identify when bias may have influenced interpretation or response, transforming awareness into action (Galvan and Payne 2024).
- Commit to intersectional practice. Recognize that race, gender, class, and other identities compound stigma, especially for Black and Brown women who use cannabis for mental health. Justice-oriented clinicians consider these intersections when assessing risk, safety, and support.

These steps establish bias-reduction as a fundamental part of professional competence—crucial for building equitable therapeutic relationships and promoting harm-reduction ethics in clinical care. These strategies build a therapeutic alliance based on transparency, respect, and mutual trust. Instead of emphasizing compliance or abstinence, clinicians collaborate with patients to minimize potential harms and improve overall wellness, one decision at a time.

#### Limitations

This Perspective is grounded in clinical experience, interdisciplinary research, and established theoretical frameworks rather than original empirical data. As such, the insights offered are interpretive and practice-oriented. The examples described are drawn from composite clinical observations and may not reflect the full diversity of patient experiences across settings and cultures. Additionally, the discussion centers primarily on U.S. policy and healthcare contexts, which may limit its direct applicability elsewhere. Future research should explore how clinicians implement justice-oriented and harm-reduction approaches in diverse populations and the measurable effects of bias-reduction strategies on clinical outcomes. Despite these limitations, this Perspective seeks to advance an ongoing conversation about equity, integrity, and compassion in mental health practice.

## Conclusion

Against this backdrop of systemic inequity, clinicians have both the opportunity and the ethical responsibility to create new narratives of safety and inclusion. One way to begin this work is by explicitly stating that conversations about cannabis are welcome. By creating a space free from judgment and stigma, patients feel empowered to speak openly about their experiences. Even so, some may begin cautiously, shaped by prior experiences where disclosure was met with silence, dismissal, or moral judgment. Others may gradually share more over time, revealing that they use cannabis to sleep, manage anxiety, or ease the weight of depression. For many, cannabis is a form of self-directed care that soothes parts of themselves that feel overwhelmed or unmanageable.

Yet these vital conversations are too often shut down in clinical spaces. Clinicians may ignore or discourage cannabis use without deeper inquiry or impose rigid expectations of abstinence. This erasure contributes to a disconnect between patients’ lived experiences and clinicians’ training, comfort, and cultural competence (Reid 2020). This silence stems from clinical education that has largely excluded cannabis, (Findley et al. 2021; Evanoff et al. 2017) from policies that have criminalized it—particularly in Black and Brown communities (Simmons 2018) —and from lingering professional discomfort (Hoffman et al. 2016; Hagiwara et al. 2020).

Clinicians are not required to endorse cannabis use, but we are ethically obligated to engage with patients with nuance, honesty, and cultural humility (Tervalon and Murray-García 1998). Rather than judging or avoiding cannabis discussions, clinicians can explore how it functions in patients’ lives, how it intersects with trauma, and how it aligns with therapeutic goals. We can begin by asking, listening, learning, and unlearning. We can shift from avoidance to engagement, from stigma to safety.

Justice-oriented practice, however, demands more than openness—it requires accountability. Meeting patients where they are must also include examining where *we* stand: our biases, privileges, and participation in systems that have historically pathologized communities of color. Critical Race Theory and Empowerment Theory remind us that equitable care begins with self-reflection, awareness of power, and action toward change (Zimmerman 2000; 2017). This work is not a one-time intervention but a continuous practice of learning, repairing, and recommitting to justice.

As clinicians, we have the power to shape whether our spaces perpetuate stigma or cultivate safety. Our willingness to confront discomfort, seek education, and center equity in clinical dialogue will define the future of mental health care. We do not need all the answers to begin these conversations—only the humility to listen, the courage to change, and the integrity to act with compassion and justice.

## Data Availability

Not applicable.

## Abbreviations

PTSD: Post-Traumatic Stress Disorder
THC: Tetrahydrocannabinol
CBD: Cannabidiol
